# LETM1 in mitochondrial cation transport

**DOI:** 10.3389/fphys.2014.00083

**Published:** 2014-02-26

**Authors:** Karin Nowikovsky, Paolo Bernardi

**Affiliations:** ^1^Department of Internal Medicine 1, Anna Spiegel Center of Translational Research, Medical University of ViennaWien, Austria; ^2^Department of Biomedical Sciences, University of PadovaPadova, Italy

**Keywords:** mitochondria, LETM1, cation transport proteins, Wolf-Hirschhorn Syndrome

LETM1 (leucine zipper- EF hand-containing transmembrane 1) encodes a highly conserved eukaryotic protein of the mitochondrial inner membrane which is essential to control mitochondrial volume homeostasis. Indeed, LETM1 RNA interference causes severe mitochondrial changes that include massive matrix swelling, loss of cristae structure and network fragmentation in *S. cerevisiae* (Nowikovsky et al., 2004), *C. elegans* (Hasegawa and van der Bliek, 2007), human cell cultures (Dimmer et al., 2008), *D. melanogaster* (McQuibban et al., 2010), and *T. brucei* (Hashimi et al., 2013). Conversely, its overexpression induces mitochondrial contraction and cristae condensation (Hasegawa and van der Bliek, 2007). LETM1 is associated with the Wolf-Hirschhorn Syndrome (WHS), a complex multigenic disease caused by haploinsufficiency of the WHSCR 1 and 2 regions on chromosome 4 (Endele et al., 1999). LETM1 is located less than 80 kb distal to WHSCR1 within the deleted locus in patients with seizures, and is preserved in all patients without seizures (Schlickum et al., 2004) implying LETM1 (hence mitochondrial dysfunction) in the pathogenesis of seizures.

LETM1 has been originally proposed to be part of the mitochondrial K^+^-H^+^ exchanger (KHE) (Nowikovsky et al., 2004; Hasegawa and van der Bliek, 2007; Dimmer et al., 2008; McQuibban et al., 2010; Hashimi et al., 2013), an essential element of Mitchell's chemiosmotic theory (Mitchell, 1966, 2011). The existence of a H^+^ electrochemical gradient driving electrophoretic K^+^ uptake indeed demands the existence of an electroneutral KHE to extrude K^+^ and thus, prevent osmotic burst of the organelle that would follow K^+^ accumulation (Mitchell, 1966, 2011). Strong support for a direct role of LETM1 in mitochondrial K^+^ homeostasis comes from the observation that inactivation of LETM1 (i) causes an increase of mitochondrial volume that can be reverted by nigericin (an ionophore that catalyzes the electroneutral exchange of H^+^ with K^+^) and (ii) can be phenocopied by the addition of valinomycin (an ionophore that catalyzes the electrophoretic flux of K^+^) (Nowikovsky et al., 2004; Froschauer et al., 2005; Dimmer et al., 2008; McQuibban et al., 2010; Hashimi et al., 2013). The idea that LETM1 catalyzes KHE (by itself or in combination with other proteins) was challenged by a genome-wide siRNA screening in Drosophila reporting that treatment with siRNA against LETM1 caused decreased Ca^2+^ influx into energized mitochondria; and that Ca^2+^ flux (proposed to be mediated by LETM1) was inhibited by ruthenium red (RR) (Jiang et al., 2009), the classical inhibitor of the mitochondrial Ca^2+^ uniporter, MCU (Moore, 1971). Based on these studies, it was suggested that LETM1 is a H^+^-Ca^2+^ antiporter catalyzing Ca^2+^ uptake in energized mitochondria (Jiang et al., 2009). As we have discussed in detail elsewhere, a H^+^-Ca^2+^ antiporter can catalyze Ca^2+^ uptake only if the H^+^/Ca^2+^ stoichiometry is lower than 2 (i.e., if net charge translocation takes place) while an antiporter with a stoichiometry of 2H^+^/Ca^2+^ can only catalyze Ca^2+^ efflux in energized mitochondria (Nowikovsky et al., 2012). A recent study bears on each of the questions outlined above (Tsai et al., 2014).

LETM1-mediated Ca^2+^ transport was assessed by applying an inward Ca^2+^ gradient across liposomes containing highly purified recombinant LETM1, and then measuring Ca^2+^ flux either with a fluorescent indicator that had been trapped inside the liposomes, or with tracer ^45^Ca^2+^accumulation (Tsai et al., 2014). Using these approaches, LETM1-dependent Ca^2+^ flux was observed when a minimum of 30 μM Ca^2+^ was added outside the liposomes. Ca^2+^ flux was insensitive to valinomycin, suggesting that it was not affected by the membrane potential and therefore electroneutral; it was stimulated by a pH gradient, while other monovalent cations were ineffective suggesting that Ca^2+^ flux occurs in exchange for H^+^; and it was insensitive to RR (Tsai et al., 2014). These findings demonstrate that the RR-sensitive Ca^2+^ uptake reported previously (Jiang et al., 2009) could not be mediated by LETM1, and suggest that impaired Ca^2+^ uptake after LETM1 knock down was an indirect consequence of mitochondrial dysfunction and deenergization, an issue on which we will return later.

The study of Tsai et al. also addressed the question of whether K^+^ is transported. LETM1-containing liposomes were loaded with K^+^ to obtain a 1000-fold outward K^+^ gradient, and then exposed to a trace amount of ^86^Rb^+^ which was not accumulated. The assumption was that an electroneutral KHE should also exchange K^+^ for K^+^ (Tsai et al., 2014), yet under the conditions of the experiment ^86^Rb^+^ distribution must follow that of K^+^, and the cation will be accumulated only if transport is electrophoretic. In other words, we believe that under the conditions of Tsai et al. LETM1 cannot catalyze KHE, and that to assess its occurrence a pH gradient should be imposed to the liposomes. The fact that both valinomycin and nigericin allowed ^86^Rb^+^ uptake into the liposomes (Tsai et al., 2014) adds to the problem, because with a 1000-fold outward K^+^ gradient ^86^Rb^+^ accumulation should be seen with valinomycin (which catalyzes electrophoretic K^+^/Rb^+^ transport) but not with nigericin which catalyzes strictly electroneutral K^+^/Rb^+^-H^+^ exchange, and would therefore require a pH gradient to catalyze ^86^Rb^+^ accumulation. Thus, we think that the study of Tsai et al. has not provided an answer as to whether LETM1 mediates KHE in a reconstituted system.

There is no question that LETM1 inactivation causes severe mitochondrial dysfunction with depolarization and matrix swelling (Nowikovsky et al., 2004; Hasegawa and van der Bliek, 2007; Dimmer et al., 2008; McQuibban et al., 2010; Hashimi et al., 2013). Matrix swelling can be easily explained if LETM1 catalyzes KHE, because its inactivation would cause excessive matrix K^+^ uptake; but how could mitochondrial dysfunction and swelling be caused by inactivation of a 2H^+^-Ca^2+^ exchanger mediating Ca^2+^ efflux? Mitochondrial Ca^2+^ distribution is governed by the balance of electrophoretic Ca^2+^ uptake through the MCU (Baughman et al., 2011; De Stefani et al., 2011) and by Ca^2+^ release through the 3Na^+^-Ca^2+^ exchanger NCLX (Palty et al., 2010) and through a still unidentified Na^+^-insensitive Ca^2+^ release system, possibly a H^+^-Ca^2+^ antiporter, which is insensitive to RR (reviewed in Nowikovsky et al., 2012). In principle, inactivation of Ca^2+^ release in mammalian mitochondria could lead to Ca^2+^ overload and possibly to an increased open probability of the permeability transition pore (PTP), a high-conductance channel which forms from dimers of the F-ATP synthase (Giorgio et al., 2013) and mediates depolarization and mitochondrial swelling [reviewed in Bernardi (2013)]. Yet, operation of the ubiquitous NCLX should easily compensate for the absence of H^+^-Ca^2+^ exchange. Thus, at present there is a solid mechanism to explain mitochondrial dysfunction following LETM1 inactivation only if LETM1 takes part in KHE.

The LETM1 gene has been evolutionarily conserved from yeast to trypanosomes, to Drosophila and mammals (Nowikovsky et al., 2004; Hasegawa and van der Bliek, 2007; Dimmer et al., 2008; McQuibban et al., 2010; Hashimi et al., 2013), suggesting that is serves a common function in these organisms. Yeast mitochondria do not possess an MCU, and therefore they cannot catalyze rapid Ca^2+^ uptake (Carafoli and Lehninger, 1971). It appears thus difficult to envision why yeast mitochondria should have developed a Ca^2+^ release mechanism which can only lead to matrix Ca^2+^ depletion. Since yeast mitochondria undergo *in situ* swelling when LETM1 is inactivated -an event that cannot be ascribed to Ca^2+^ overload- we still favor the hypothesis that LETM1 catalyzes KHE *in situ*, as overwhelming evidence suggests (Nowikovsky et al., 2004; Hasegawa and van der Bliek, 2007; Dimmer et al., 2008; McQuibban et al., 2010; Hashimi et al., 2013). Can these apparently conflicting sets of data be reconciled? It is possible that cation selectivity *in situ* is affected by protein interactions that are lost in the reconstituted system, and thus that LETM1 catalyzes RR-insensitive, electroneutral exchange of H^+^ with both K^+^ and Ca^2+^, an issue that should be addressed in future studies of this fascinating problem.
